# Cephalosporin as Potent Urease and Tyrosinase Inhibitor: Exploration through Enzyme Inhibition, Kinetic Mechanism, and Molecular Docking Studies

**DOI:** 10.1155/2022/1092761

**Published:** 2022-07-28

**Authors:** Yahya S. Alqahtani, Bandar A. Alyami, Ali O. Alqarni, Mater H. Mahnashi, Anser Ali, Qamar Javed, Mubashir Hassan, Muhammad Ehsan

**Affiliations:** ^1^Department of Pharmaceutical Chemistry, College of Pharmacy, Najran University, Najran, Saudi Arabia; ^2^Department of Zoology, Mirpur University of Science and Technology (MUST), Mirpur, 10250 AJK, Pakistan; ^3^The Steve and Cindy Rasmussen Institute for Genomic Medicine, Nationwide Children's Hospital, Columbus, Ohio 43205, USA; ^4^Department of Chemistry, Mirpur University of Science and Technology (MUST), Mirpur, 10250 AJK, Pakistan

## Abstract

In present study, eleven cephalosporin drugs were selected to explore their new medically important enzyme targets with inherited safety advantage. To this end, selected drugs with active ingredient, cefpodoxime proxetil, ceftazidime, cefepime, ceftriaxone sodium, cefaclor, cefotaxime sodium, cefixime trihydrate, cephalexin, cefadroxil, cephradine, and cefuroxime, were evaluated and found to have significant activity against urease (IC50 = 0.06 ± 0.004 to 0.37 ± 0.046 mM) and tyrosinase (IC50 = 0.01 ± 0.0005 to 0.12 ± 0.017 mM) enzymes. Urease activity was lower than standard thiourea; however, tyrosinase activity of all drugs outperforms (ranging 6 to 18 times) the positive control: hydroquinone (IC50 = 0.18 ± 0.02 mM). Moreover, the kinetic analysis of the most active drugs, ceftriaxone sodium and cefotaxime sodium, revealed that they bind irreversibly with both the enzymes; however, their mode of action was competitive for urease and mixed-type, preferentially competitive for tyrosinase enzyme. Like *in vitro* activity, ceftriaxone sodium and cefotaxime sodium docking analysis showed their considerable binding affinity and significant interactions with both urease and tyrosinase enzymes sufficient for downstream signaling responsible for observed enzyme inhibition *in vitro*, purposing them as potent candidates to control enzyme-rooted obstructions in future.

## 1. Introduction

The cephalosporins are common antibiotics prescribed in routine for broad range of infections. Lesser toxic and allergic threats along with wide action spectrum make them popular [1]. They possess *β*-lactam ringed structure similar to penicillin. This interferes with the synthesis of bacterial cell wall showing significant antibacterial properties. Guiseooe Brotzu, Italian scientist, isolated cephalosporin compounds from Cephalosporium acremonium cultures in 1948 [2]. They are classified generation wise, lower generations possess strong activity against gram-positive bacteria, and higher generations possess more activity against gram-negative bacteria; however, cefepime from fourth generation possesses both gram-positive activity (equivalent to first generation) and gram-negative activity (equivalent to third generation) [3]. Third generation cephalosporins are active against gram-negative rods, especially Enterobacter and multiple resistant strains. They are proven helpful in controlling hospital-acquired infections including bacteremia and pneumonia [2]. For present study, eleven drugs from cephalosporin class with single active compound, cefpodoxime proxetil, ceftazidime, cefepime, ceftriaxone sodium, cefaclor, cefotaxime sodium, cefixime trihydrate, cephalexin, cefadroxil, cephradine, and cefuroxime, were purchased aiming to explore their potential against biologically important two enzymes, urease and tyrosinase.

Urease, a nickel-dependent thiol-rich metalloenzyme is responsible for ammonia and carbamate formation from urea [4]. It is usually present in bacteria, fungi, algae, plants, and invertebrates. It is also present in soil as a soil enzyme [5]. The important components of ureases for catalytic activity are Ni^2+^ ions and the sulfhydryl group (especially the cysteinyl residues in the active site). An important virulence factor of many bacterial species including *Klebsiella pneumoniae*, *Proteus mirabilis*, *Salmonella* species, *Staphylococcus* species, and *Ureaplasma urealyticum* is their ureolytic activity. It is associated with pathogenesis of certain medical conditions, i.e., hepatic coma, pyelonephritis, urinary stone formation, and peptic ulceration [6, 7]. Increased pH (up to 9.2) during hydrolyses of urea is observed [6]. Thus, urease activity helps bacteria to adjust pH allowing them to survive even in originally low pH of stomach causing stomach cancer and peptic ulcers during colonization [8]. Hence, urease inhibitors are the first-line strategy to control infections caused by urease-producing microorganisms.

Tyrosinase, our second study enzyme, is associated with melanin synthesis responsible for hair and skin colour [9, 10]. Melanin is formed from L-tyrosine conversion into 3,4-dihydroxyphenylalanine (L-DOPA) which oxidizes to produce dopaquinone [11]. Thus, the tyrosinase enzyme regulates the melanin content which protects skin from UV radiations and sun burn. However, its overexpression results in hyperpigmentation causing dermatological disorders, i.e., melisma and age spots [12]. Moreover, neuromelanin in the brain and neurodegeneration are known to be linked with Parkinson's disease [13]. Tyrosinase induction produces reactive oxygen species known to cause neurotoxicity [14]. Thus, discovery of tyrosinase inhibitors is important for tyrosinase control and treatment of melanin-related skin complications [15, 16]. Although many tyrosinase inhibitors are identified however, their toxic effects prohibit their commercialization, indicating the need to search new safe and effective alternatives.

Thus, the focus of study is to use already existing safety proven drugs to explore their new therapeutic targets, an effective strategy which not only allow to maximize the use of drug's potential but also help to reduce evaluation time, cost, and risk of failure. Thus, eleven cephalosporin drugs were selected to evaluate their potential against two medically important enzymes. Later, kinetic study of two most potent drugs was executed and evaluated their kinetic parameters and inhibition constants to explore their mechanism of enzyme inhibition. Moreover, a plot among remaining enzyme activity versus various concentrations of respective enzymes in the presence of selected drugs was devised as determinant of reversible or irreversible behaviour of enzyme inhibition. Finally, docking study identifying the binding pattern of drug with enzyme which is important for observed enzyme inhibition was executed.

## 2. Materials and Methods

### 2.1. Chemicals

Enzymes, mushroom tyrosinase, and urease were purchased from Sigma. Eleven drugs from cephalosporin class were purchased from local pharmacy, and their active ingredients were summarized in Table 1 and Figure S1 with formula [17–27]. To prepare stock, ground powder was weighted to directly dissolve in DMSO. All items were stored in recommended conditions with shelf life of safe use till all evaluations.

### 2.2. Urease Inhibitory Assay

To evaluate the urease enzyme activity, assay described by Weatherburn 1967 was performed [28]. In 96-well plate, 10 *μ*l of enzyme (jack bean urease, 5 U/ml), 40 *μ*l buffer (100 mM urea, 0.01 M K2HPO4, 1 mM EDTA, and 0.01 M LiCl2, pH 8.2), and 20 *μ*l of test drug were loaded. Following 15 min incubation at 37°C, 40 *μ*l of alkali reagent (0.5%, *w*/*v* NaOH and 0.1% active chloride NaOCl) and 40 *μ*l of phenol reagents (1%, *w*/*v* phenol and 0.005%, *w*/*v* sodium nitroprusside) were added. After 35 min incubation at room temperature (RT), OD625 nm was tracked to calculate IC50 values to compare the test drugs result with standard named thiourea.

### 2.3. Tyrosinase Inhibitory Assay

To evaluate tyrosinase inhibition, assay was performed as described previously [29]. Reaction was started by loading 140 *μ*l of phosphate buffer (20 mM, pH 6.8), 20 *μ*l of mushroom tyrosinase (30 U/ml), and 20 *μ*l of test drug in 96-well plate. After 10 min incubation at RT, 20 *μ*l (0.85 mM) L-DOPA (3,4-dihydroxyphenylalanine) was added and incubated again for 20 min at RT. Then, OD475 nm was determined as measure of dopachrome formation by plate reader (BioTek, Elx 800). Kojic acid was used as standard inhibitor for reference. For clear statistical analysis, experiments were performed twice in duplet. First percentage inhibition was determined and then IC50 was calculated using Microsoft excel, and the test drug results were compared with standard.

### 2.4. Study of Enzyme Kinetics

To evaluate the type of enzyme inhibition, series of kinetic experiments were performed using 2 most active drugs against both enzymes, urease and tyrosinase, following methods reported previously [29, 30]. To this end, the Lineweaver-Burk plots of 1/absorbance versus 1/urea and 1/absorbance versus 1/L-DOPA were plotted. In all kinetic studies, drug concentrations (as indicated in Lineweaver-Burk plot) and respective substrates, urea in buffer (0.063 to 2 mM) for urease and L-DOPA (0.06 to 2 mM) for tyrosinase, were added and plates were incubated for 10 min at 37°C. Later, respective enzymes were added in plates and absorbance (wavelengths same as above) was monitored for 5 min with 1 min interval. The Lineweaver-Burk plot showing type of enzyme inhibition was plotted as inverse of velocities (1/V) versus inverse of substrate concentration 1/[S] Mm^−1^. Later, inhibition constant (Ki) was evaluated by both the Dixon plot and from Lineweaver-Burk plot, by secondary replot of slope versus concentrations of inhibitor.

### 2.5. Inhibition Mechanism of Potential Inhibitor

The inhibitory mechanism of both the enzymes, urease and tyrosinase, was determined with two most active drugs following Tahir et al. and Ali et al. [30, 31]. To this end, a plot among remaining enzyme activity versus various concentrations of respective enzymes in the presence of drug concentrations (as indicated in graph) was devised as determinant of reversible or irreversible behaviour of enzyme inhibition.

### 2.6. In Silico Study: Repossession of Jack Bean Urease and Mushroom Tyrosinase from PDB

The crystal structures of jack bean urease and mushroom tyrosinase were retrieved from the Protein Data Bank (PDB) having PDBIDs 4H9M and PDBID 2Y9X (http://www.rcsb.org/), respectively. Furthermore, energy minimization of target, stereochemical properties, Ramachandran graph, and values of urease and mushroom tyrosinase were explored [32, 33].

Moreover, to access architecture of study proteins and occurrence of *α*-helices, *β*-sheet and coil tool called VADAR 1.8 was used (http://vadar.wishartlab.com/).

### 2.7. Designing of Ligands and Molecular Docking Simulation Using Autodock

The drug molecules cefpodoxime proxetil, ceftazidime, cefepime, ceftriaxone sodium, cefaclor, cefotaxime sodium, cefixime trihydrate, cephalexin, cefadroxil, cephradine, and cefuroxime were sketched in drawing ACD/ChemSketch tool and further minimized by visualizing software UCSF Chimera 1.10.1. PyRx docking tool was used to perform molecular docking experiment for the ligands against urease and tyrosinase enzymes [34]. The grid box center values of urease were adjusted as center_ *X* = 18.0279, *Y* = −57.332 and *Z* = −18.5254, and for tyrosinase, it was fixed as center_ *X* = −12.385, *Y* = −18.7636, and Z = −46.7393, respectively, for better conformational position in the active region of target proteins. The selected drugs were docked with default exhaustiveness value = 8, and resultant complexes were evaluated on the basis of lowest binding energy (Kcal/mol) and structure activity relationship (SAR). The three-dimensional (3D) graphical depictions of all the docked complexes were accomplished by Discovery Studio (2.1.0) (https://discover.3ds.com/discovery-studio-visualizer-download) and UCSF Chimera 1.10.1 [32].

## 3. Results and Discussion

In present study, we selected eleven antibiotics from cephalosporin family aiming to maximize the use of their potential for multiple applications with inherited safety advantages and rooting out their new biological targets such as enzymes, urease and tyrosinase, with possible inhibition mechanism eventually proposing effective and safe alternative for the management of enzyme-associated medical obstructions.

Our results confirmed that drugs with active ingredients, cefpodoxime proxetil, ceftazidime, cefepime, ceftriaxone sodium, cefaclor, cefotaxime sodium, cefixime trihydrate, cephalexin, cefadroxil, cephradine, and cefuroxime, showed excellent activity against urease and tyrosinase enzymes with 50% inhibitory concentration (IC50) ranging from 0.06 ± 0.004 to 0.22 ± 0.006 mM and 0.01 ± 0.0005 to 0.12 ± 0.017 mM, respectively. Urease activity of all drugs was noted lower than positive control thiourea (IC50 = 0.019 ± 0.002 mM); however, tyrosinase activity of all drugs outperforms the positive controls: hydroquinone (IC50 = 0.18 ± 0.02 mM). Cefotaxime sodium and ceftriaxone sodium showed lower IC50 among all test drugs for both urease (IC50 = 0.06 and 0.08 mM, respectively) and for tyrosinase (IC50 = 0.01 and 0.03 mM, respectively).

In other words, ceftriaxone sodium and cefotaxime sodium showed 18 and 6 times better tyrosinase activity than standards hydroquinone.

In biological reactions, enzymes play key role and therefore are considered attractive target in disease control [31, 35]. Likewise tyrosinase, being the rate-limiting player in darkening of skin and fruits, its inhibition is desirable both in cosmetics and food industry. Multiple depigmenting agents called inhibitors such as arbutin [36], azelaic acid [37], retinoids [38], ascorbic acid derivatives [39], kojic acid [40], and hydroquinone [41] are known. However, unwanted side effects including cytotoxicity are observed from many well-known whitening agents such as hydroquinone and kojic acid which minimizes their use. Interestingly, all tested drugs showed activity; however, cefotaxime sodium and ceftriaxone sodium showed multifold better tyrosinase inhibitory effect than standard hydroquinone. Thus, to understand the mechanism of observed enzyme inhibition, study of enzyme kinetics was performed.

### 3.1. Mechanism of Urease Enzyme Kinetics

**Figure 1:**
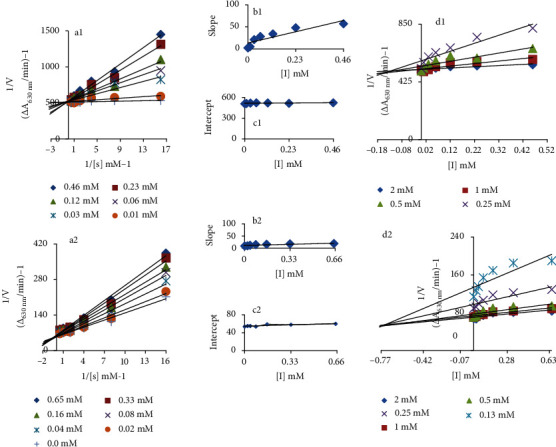
(a1 and a2) Lineweaver-Burk plot for inhibition of urease enzyme in the presence of cefotaxime sodium and ceftriaxone sodium. The cefotaxime sodium concentrations 0, 0.01, 0.03, 0.06, 0.12, 0.23, and 0.46 mM and ceftriaxone sodium concentrations 0, 0.02, 0.04, 0.08, 0.16, 0.33, and 0.65 mM; however, urea concentrations ranging from 0.13 to 2 mM were used. (b1 and b2) The insets represent the plot of the slope from the Lineweaver-Burk plot versus inhibitor. (c1 and c2) The secondary replot of the Lineweaver-Burk plot, 1/V (y-intercept) of (a) versus various concentrations of inhibitor. (d1 and d2) The Dixon plot of the reciprocal of the initial velocities versus various concentrations of inhibitor.

To understand the mechanism of urease inhibition, series of kinetic experiments against two most active drugs, cefotaxime sodium and ceftriaxone sodium, were performed and the respective Lineweaver-Burk and Dixon plots were generated (Figures 1(a1) and 1(a2)). The Lineweaver-Burk plots, *1/V* versus 1/[S], follows Michaelis-Menten kinetics and showed that both drugs behave as competitive inhibitor since increase in their concentration produced a family of straight lines with a common intercept on the ordinate but with different slopes [42]. To obtain insightful pathway, binding affinities of EI and ESI complexes were determined. Analysis revealed competitive mode of urease inhibition (Figures 1(a1) and 1(a2)). The secondary replots of slope versus drug concentration and secondary replots of intercept versus drugs concentration showed EI dissociation constant (Ki) (Figures 1(b1) and 1(b2)) and ESI dissociation constant (Ki') (Figures 1(c1) and 1(c2)). The *Ki* values for cefotaxime sodium and ceftriaxone sodium were calculated 0.12 and 0.7 mM, respectively, by both the Dixon plot and secondary replot from the Lineweaver-Burk plot of slope. However, *Ki'* values, 30 mM (cefotaxime sodium) and 6 mM (ceftriaxone sodium), were determined by secondary replot of the Lineweaver-Burk plot of intercept. Comparison showed less *Ki* compared to *Ki'* values indicating stronger binding between enzyme and drug [43] justifying preferred competitive mode of inhibition.

### 3.2. The Inhibitory Effect of Drugs on Urea Hydrolysis Activity of Urease

To further understand the urease reversible or irreversible inhibitory behaviour by ceftriaxone sodium and cefotaxime sodium, experiments were performed as described in Materials and Methods.

**Figure 2:**
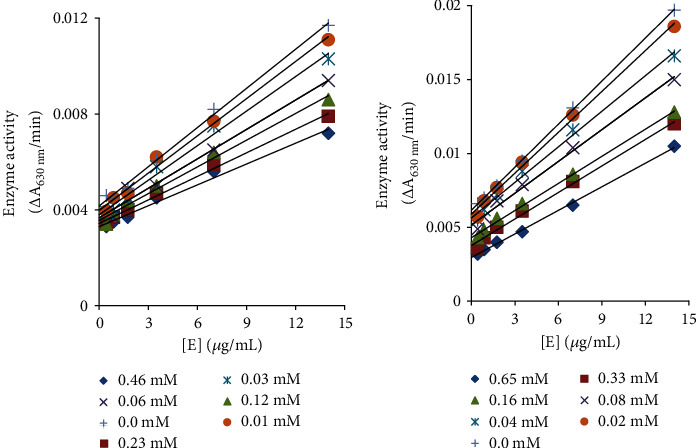
Catalytic activity relationship of urease and various concentrations of (a) cefotaxime sodium and (b) ceftriaxone sodium.

Plots among enzyme activity versus the concentration of enzyme (0.44, 0.88, 1.75, 3.5, 7, and 14 *μ*g/ml) in the presence of drugs produced a group of straight lines (Figure 2). These parallel straight lines with the same slopes indicate irreversible urease inhibition [44, 45]. Thus, our both drugs, ceftriaxone sodium and cefotaxime sodium, are shown to bind effectively with urease active site to inhibit irreversibly.

### 3.3. Mechanism of Tyrosinase Enzyme Kinetics

**Figure 3:**
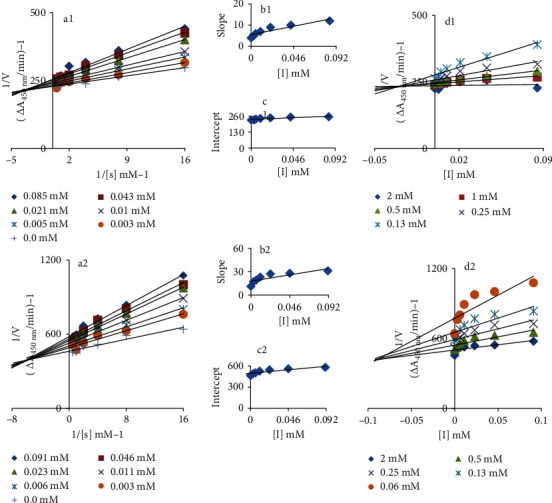
(a1 and a2) Lineweaver-Burk plot for inhibition of tyrosinase enzyme in the presence of cefotaxime sodium and ceftriaxone sodium. The cefotaxime sodium concentrations 0, 0.008, 0.02, 0.03, 0.07, 0.14, and 0.27 mM and ceftriaxone sodium concentrations 0, 0.003, 0.006, 0.011, 0.023, 0.046, and 0.091 mM; however, L-DOPA concentrations ranging 0.06 to 2 mM were used. (b1 and b2) The insets represent the plot of the slope from the Lineweaver-Burk plot versus inhibitor. (c1 and c2) The secondary replot of the Lineweaver-Burk plot, 1/V (y-intercept) of (a) versus various concentrations of inhibitor. (d1 and d2) The Dixon plot of the reciprocal of the initial velocities versus various concentrations of inhibitor.

The mode of tyrosinase inhibition against two most active drugs, ceftriaxone sodium and cefotaxime sodium was determined by tracking oxidation of L-DOPA through the Lineweaver-Burk and Dixon plots. In the Lineweaver-Burk plots, *1/V* versus 1/[S] produced a family of different straight slopes (Figures 3(a1) and 3(a2)). Evaluation showed that *V*max reduces with *Km* shift and increasing concentrations of ceftriaxone sodium and cefotaxime sodium, revealing their mixed type inhibitory behaviour. This means that drugs can interact with free enzyme (E) and enzyme-substrate (ES) complex [46]. To obtain insightful pathway, binding affinities of EI and ESI complexes were determined. The secondary replots for EI dissociation constant (Ki) (Figures 3(b1) and 3(b2)) and ESI dissociation constant (Ki') (Figures 3(c1) and 3(c2)) extracted. The values of *Ki* and *Ki'* were calculated as 0.1 and 0.6 mM (ceftriaxone sodium) and 0.07 and 0.8 mM (cefotaxime sodium), respectively. Comparison showed less *Ki* compared to *Ki'* values indicating stronger binding between enzyme and drug [43] that indicate preferentially competitive in mixed type mode of enzyme inhibition.

### 3.4. The Inhibitory Effect of Drugs on Diphenolase Activity of Tyrosinase

**Figure 4:**
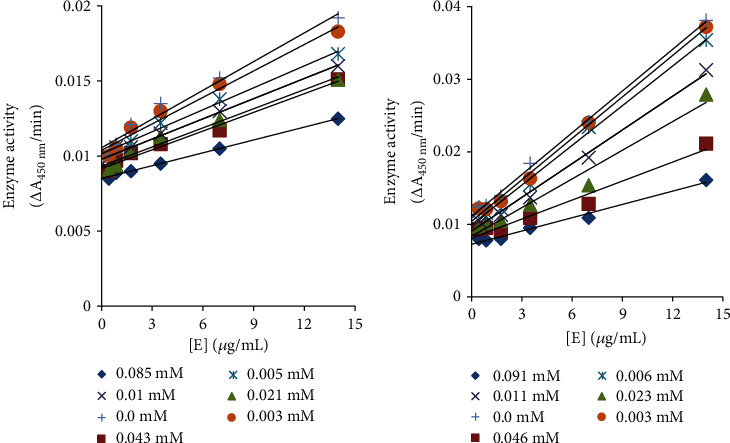
Catalytic activity relationship of tyrosinase and various concentrations of (a) cefotaxime sodium and (b) ceftriaxone sodium.

To explore mechanism further and tyrosinase reversible or irreversible inhibitory behaviour, diphenolase activity of both drugs ceftriaxone sodium and cefotaxime sodium was performed. Plots among enzyme activity versus the concentration of enzyme (0.44, 0.88, 1.75, 3.5, 7, and 14 *μ*g/ml) in the presence of different concentrations of drugs a family of straight lines were generated (Figure 4). The parallel straight lines with the same slopes indicate irreversible mode of enzyme inhibition [44, 45]. Thus, like urease, both the most potent drugs, ceftriaxone sodium and cefotaxime sodium ,were irreversible inhibitors of mushroom tyrosinase for oxidation of L-DOPA.

### 3.5. Structural Assessment of Target Proteins

Urease (Jack bean) have tetra domains with different numbers of residues. Among all, the most important is the domain four due to presence of binding pocket and its catalytic behaviour. It consists 27% *α*-helices, 31% *β*-sheets, and 41% coils. The Ramachandran plots showed occurrence of 97.5% residues in favored regions evolving phi (*φ*) and psi (*ψ*) angle's good precision among the coordinates of jack bean urease structure (Figure S2).

Second enzyme, mushroom tyrosinase oxidoreductase copper, contains enzyme consisting of 391 amino acids with structural contribution of 39% *α*-helices (154 residues), 14% *β*-sheet (57 residues), and 46% coil (180 residues). Its resolution 2.78 Å, *R* value 0.238, and unit cell length as *a* = 103.84, *b* = 104.82, and *c* = 119.36 with angles 90°, 110.45°, and 90° for all *α*, *β*, and *γ* dimensions were observed, respectively. The Ramachandran plots verified the occurrence of 95.90% residues in favored and 100.0% residues in allowed regions. The Ramachandran graph displayed good accuracy of phi (*φ*) and psi (*ψ*) angles among the coordinates of receptor molecules and most of residues plummeted in acceptable region (Figure S2).

### 3.6. Molecular Docking Analysis

The docked complexes of cefpodoxime proxetil, ceftazidime, cefepime, ceftriaxone sodium, cefaclor, cefotaxime sodium, cefixime trihydrate, cephalexin, cefadroxil, cephradine, and cefuroxime against study enzymes were evaluated based on minimum energy values (Kcal/mol) and ligand interactions pattern. The docking energy values of jack bean urease and mushroom tyrosinase docked complexes have been tabulated in Table 1.

#### 3.6.1. Binding Analyses of Drugs against Jack Bean Urease

**Figure 5:**
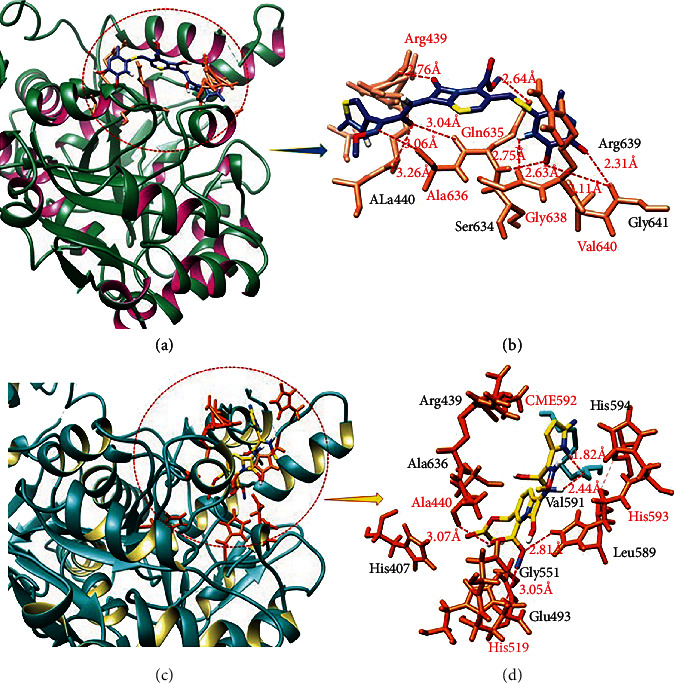
Ceftriaxone sodium (a and b) and cefotaxime sodium (c and d) binding analysis against urease.

Ceftriaxone sodium: the ligand-protein binding analyses showed that ceftriaxone sodium confined in the active binding pocket of target protein as mentioned in Figures 5(a) and 5(b). The results of ceftriaxone sodium-jack bean urease docked complex showed nine hydrogen bonds. The oxygen atoms of ceftriaxone sodium form hydrogen bond against Arg439 with bonds length 2.76 Å and 3.26 Å, respectively; moreover, the nitrogen atom of drug forms hydrogen bond with Ala636 with the bond distance 3.26 Å. Two oxygen atoms and one nitrogen atom of ceftriaxone sodium make hydrogen bond with Gln635 having bond lengths 3.04 Å, 2.63 Å, and -2.64 Å, respectively. Another hydrogen atom of drug forms hydrogen bond with Gly638 with bond length of 2.75 Å, and two other oxygen atoms form hydrogen bond with Val640 with bond length 2.11 Å and 2.31 Å, respectively.

Cefotaxime sodium: cefotaxime sodium also found to confine in urease active region as mentioned in Figures 5(c) and 5(d). The results of cefotaxime sodium-jack bean urease docked complex showed that five hydrogen bonds depict the stability of drug against target protein. The two hydrogen atoms of cefotaxime sodium formed hydrogen bond against CME592 with bond length 2.44 Å and 1.82 Å, respectively. Another hydrogen was also observed between hydrogen atom and Ala440 with bond length 3.07 Å. Moreover, oxygen and nitrogen atoms of cefotaxime sodium also formed hydrogen bond with His593 and His519 with bond distances 2.81 Å and 3.05 Å, respectively. The other 2D depiction of urease is shown in Figure S3. The predicted results showed good correlation with published research data which strengthens our work and efficacy [47–49].

#### 3.6.2. Binding Analyses of Drugs against Mushroom Tyrosinase

**Figure 6:**
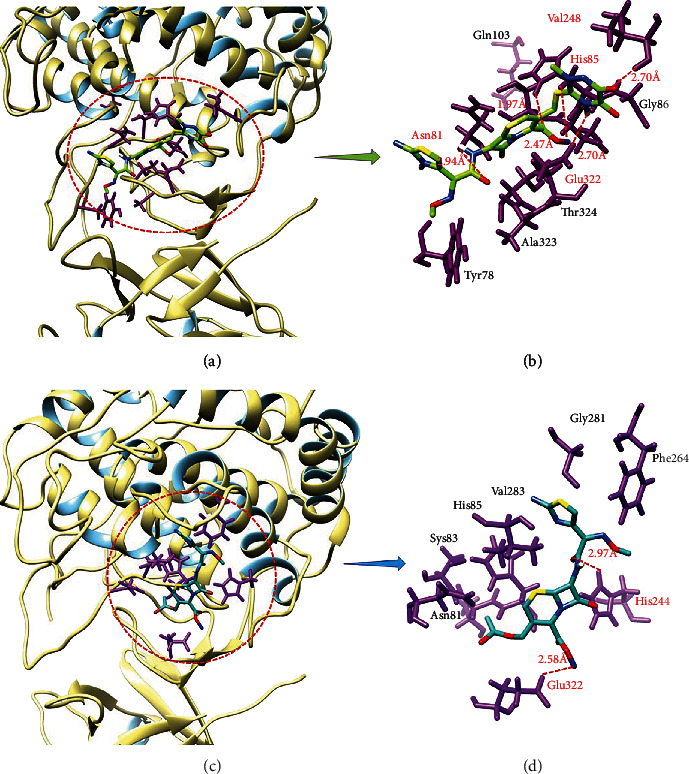
Ceftriaxone sodium (a and b) and cefotaxime sodium (c and d) binding analysis with tyrosinase.

Ceftriaxone sodium: the binding analyses of ceftriaxone sodium showed that it was confined in the active binding pocket of tyrosinase as indicated (Figures 6(a) and 6(b)). Ceftriaxone sodium-mushroom tyrosinase docked complex showed 5 hydrogen bonds. The oxygen atom of ceftriaxone sodium forms hydrogen bond against Asn81 with bond length 2.94 Å, and the nitrogen and oxygen atoms formed hydrogen bonds with His85 with the bond length of 2.47 Å and 1.97 Å, respectively. Moreover, hydrogen atom of ceftriaxone sodium formed hydrogen bond with Glu322 with bond length of 2.70 Å. Similarly, another oxygen atom of ceftriaxone sodium forms hydrogen bond with Val248 with bond length of 2.70 Å. This shows good commitment with previous literature [50–52]. Cefotaxime sodium: the ligand-protein binding analyses showed that cefotaxime sodium confined in the active binding pocket of target protein as mentioned in Figures 6(c) and 6(d). The results of cefotaxime sodium-mushroom tyrosinase docked complex showed that 2 hydrogen bonds were observed. An oxygen atom form hydrogen bond with His244 with bond length of 2.97 Å and the nitrogen atom of compound form hydrogen atom with Glu322 with bond length of 2.58 Å. The other 2D depiction of tyrosinase is shown in Figure S3. Our docking results show good correlation with published research which strengthens our work and efficacy [47, 53]. The deep interaction profiles of drugs against urease and mushroom tyrosinase clearly depicted the significance of drugs in the enzyme activity. The binding pocket residues are more important and active key players in the activation of signaling pathways [54]. In our predicted results, drugs directly interact with active site residues of both urease and mushroom tyrosinase which depicts that binding of drugs may affect the activity of enzymes and showed good correlation with *in vitro* results.

Furthermore, rest of all drugs-docking complexes have been mentioned in supporting data. Moreover, Figure S3 confirms drugs binding with urease enzyme through various amino acids; cefaclor interacts through Glu642, Gln649, Arg646, and Phe840, cefadroxil interacts through His492, His519, His593, and Arg439, cefepime interacts through Arg439, Met637, and Asp633, cefixime trihydrate interacts through His519, His492, Ala440, Arg609, and Ala636, cefpodoxime proxetil interacts through Arg439, Asp494, and Met588, and ceftazidime interacts through Ala636 and Arg439; cefuroxime interacts through Leu833, Ser579, Thr578, Arg646, and Phe838; cephalexin interacts through Arg646, Ser645, Thr581, and Phe838; cephradine interacts through Arg639, Arg646, and Glu584. Likewise, Figure S4 represents drugs binding with tyrosinase enzyme through various amino acids; cefaclor interacts through Asn260, Val283, His244, and Ala286; cefadroxil interacts through His244, His85, Met280, and His263; cefepime interacts through Ala80, Asn81, and His244; cefixime trihydrate interacts through A323, Asn81, His244, and Cys83; cefpodoxime proxetil interacts through Met319, Arg321, His244, Gly86, and Val248; ceftazidime interacts through Asn81, Cys83, His85, Glu322, Val283, and Ala286; cefuroxime interacts through Glu103, Pro329, Cys83, Asn81, and His85; cephalexin interacts through Val283, Asn81, and His85; cephradine interacts through Val283, His244, Ala323, and Asn81. Based on the results, it has been observed that both ceftriaxone sodium and cefotaxime sodium showed highest urease and tyrosinase inhibition, interestingly outperforming tyrosinase positive control, hydroquinone proposing them potential candidates to control enzyme-rooted irregularities in future.

### 3.7. Structure Activity Relationship (SAR) Analysis

The SAR is the relationship between the chemical structure having different incorporated functional groups (Figure S1) and its biological activities against different enzymes. The cefotaxime sodium, cefixime trihydrate, and cefpodoxime proxetil have basically the same skeleton with different functional groups. Similarly, the other drugs ceftriaxone sodium, cefepime, and ceftazidime correlate with each other in terms of basic structure. Cefaclor, cefadroxil, and cefuroxime are the same, while cephalexin resembles with cephradine. The different drugs showed different inhibition behaviour and docking energy values. All the compounds have potential to block the entry of substrate by binding to amino acid residues lying at the pocket domain. The enzyme/inhibitor complexes are stabilized by number of different interactions such as H-bonding, *π*-sigma interactions, *π*-alkyl interactions, *π*-anion/cation sulphur interactions, polar interactions, stacking, and metal-ligand interactions. We discuss the binding mode of two most active compounds (ceftriaxone sodium and cefotaxime sodium) and compare their interactions with the standard ligands. Figures 5 and 6 illustrate the relative positioning of ceftriaxone sodium and cefotaxime sodium in their most stable conformation with minimal energy in the active site of target. Different binding interactions were observed for ceftriaxone sodium and cefotaxime sodium due to structural differences and presence of an additional sodium citrate group in both the drugs. The ceftriaxone sodium showed good inhibition values 0.08 ± 0.004 and 0.01 ± 0.0005 (mM) with binding affinity -7.90 and -8.40 (Kcal/mol) as compared to other selected drugs (Table 1). Ceftriaxone sodium is bulky molecule containing dioxone group at one end and cyclopentane attached with amino group at another end. Moreover, sodium citrate moiety was present at the central region with benzene ring. Similarly, cefpodoxime proxetil is also a bulky structure composed different moieties such as isopropoxide, couple of methoxy and amino groups at neighboring ends which gave inhibition and docking values against urease (0.1 ± 0.014; -5.10) and mushroom tyrosinase (0.05 ± 0.0003; -5.40), respectively. Both ceftazidime and cefepime possessed acetate ions and amino group which reveals closely related inhibition and docking energy values against urease (0.091 ± 0.007; -7.50 and 0.09 ± 0.006; -7.50) and tyrosinase (0.11 ± 0.005; -7.90 and 0.19 ± 0.037; -8.90). In comparison with all other drugs, most of compounds possessed similar basic skeleton with different functional groups at different positions which depicted different inhibition and docking values against both urease and mushroom tyrosinase, respectively. Therefore, due to the presence of different functional groups in different drugs, it showed different inhibition values and binding affinities (Table 1).

## 4. Conclusion

In present study, eleven cephalosporin drugs with single active ingredient were evaluated and found to inhibit medically important both the enzymes, urease and tyrosinase *in vitro*. All drugs outperform the positive control: hydroquinone for tyrosinase activity. The kinetic analysis of most active drugs, ceftriaxone sodium and cefotaxime sodium, revealed that they bind irreversibly with both enzymes; however, their mode of action was competitive for urease and mixed-type, preferentially competitive for tyrosinase enzyme. In addition, docking study showed their significant bonding with both urease and tyrosinase enzymes purposing them potent candidates to control enzyme-rooted complications in future.

## Figures and Tables

**Table 1:** Enzyme activity of test drugs and docking energy values.

Selected drugs	Jack bean urease inhibition	Mushroom Tyrosinase inhibition	Jack bean urease docking energy	Mushroom Tyrosinase docking energy
	IC50 ± SEM (mM)		(Kcal/mol)	
Cefpodoxime proxetil	0.1 ± 0.014	0.05 ± 0.0003	-5.10	-5.40
Ceftazidime	0.091 ± 0.007	0.11 ± 0.005	-7.50	-7.90
Cefepime	0.19 ± 0.037	0.09 ± 0.006	-7.50	-8.90
Ceftriaxone sodium	0.08 ± 0.004	0.01 ± 0.0005	-7.90	-8.40
Cefaclor	0.17 ± 0.016	0.03 ± 0.005	-7.20	-7.00
Cefotaxime sodium	0.06 ± 0.004	0.03 ± 0.002	-7.50	-7.60
Cefixime trihydrate	0.18 ± 0.014	0.04 ± 0.002	-6.50	-80
Cephalexin	0.37 ± 0.046	0.12 ± 0.017	-6.30	-7.60
Cefadroxil	0.22 ± 0.006	0.03 ± 0.003	-7.20	-7.70
Cephradine	0.12 ± 0.009	0.07 ± 0.006	-6.50	-6.30
Cefuroxime	0.18 ± 0.009	0.07 ± 0.009	-6.20	-7.10
Standard	0.019 ± 0.002 Thiourea	0.18 ± 0.02 Hydroquinone	—	—

## Data Availability

The data used to support the findings of this study are included within the supplementary information file(s).

## Supplementary Materials

Supplementary Materials Figure S1: chemical structures of test drugs and resemblance of their basic inner structure and different functional groups at different locations. Figure S2: Ramachandran graph of (a) jack bean urease and (b) mushroom tyrosinase. Figure S3: 2D docking of test drugs with urease enzyme. Figure S4: 2D docking of test drugs with tyrosinase enzyme.
