# Supplementary material for: Cephalosporin as Potent Urease and Tyrosinase Inhibitor: Exploration through Enzyme Inhibition, Kinetic Mechanism, and Molecular Docking Studies
Source: Biomed Res Int. 2022 Jul 28;2022:1092761. doi: 10.1155/2022/1092761 (PMC9352478; doi:10.1155/2022/1092761)
Supplement: Supplementary Materials — Figure S1: chemical structures of test drugs and resemblance of their basic inner structure and different functional groups at different locations. Figure S2: Ramachandran graph of (a) jack bean urease and (b) mushroom tyrosinase. Figure S3: 2D docking of test drugs with urease enzyme. Figure S4: 2D docking of test drugs with tyrosinase enzyme. [file 1092761.f1.docx]

Cephalosporin as potent urease and tyrosinase inhibitor; exploration through enzyme inhibition, kinetic mechanism and molecular docking studies

**Yahya S. Alqahtani ^1^, Bandar A. Alyami ^1^, Ali O. Alqarni^1^, Mater H. Mahnashi ^1^*, Anser Ali^2^, Qamar Javed^2^, Mubashir Hassan^3^, Muhammad Ehsan^4^**

^1^Department of Pharmaceutical Chemistry, College of Pharmacy, Najran University, Najran, Kingdom of Saudi Arabia

^2^Department of Zoology, Mirpur University of Science and Technology (MUST), Mirpur-10250 (AJK)- Pakistan

^3^The Steve and Cindy Rasmussen Institute for Genomic Medicine, Nationwide Children’s Hospital, Columbus, Ohio 43205, United States

^4^Department of Chemistry, Mirpur University of Science and Technology (MUST), Mirpur-10250 (AJK)- Pakistan

* Corresponding author E-mail address: [aleen9542@gmail.com](mailto:aleen9542@gmail.com) (Mater H. Mahnashi)

FIGURE S1: Chemical structures [adapted from 17-27] of test drugs and resemblance of their basic inner structure and different functional groups at different locations.

**
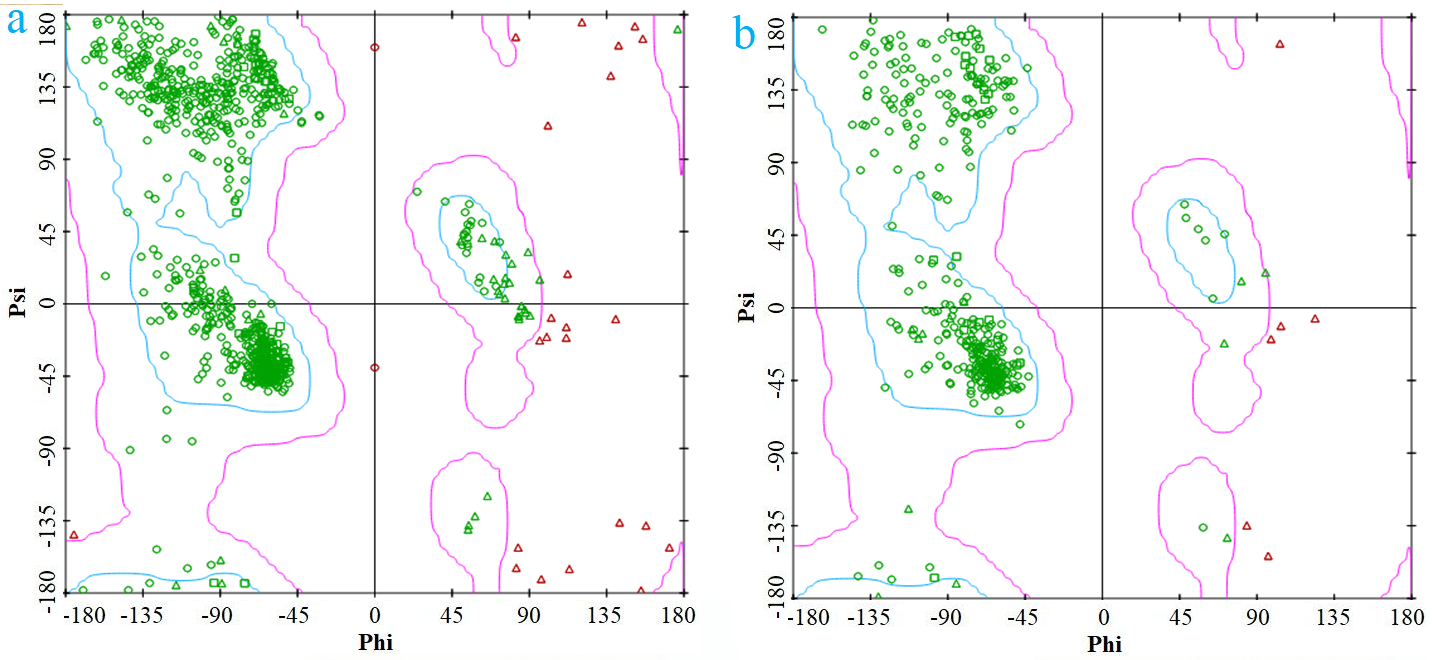
**

FIGURE S2: Ramachandran graph of (a) jack bean urease and (b) mushroom tyrosinase.

**
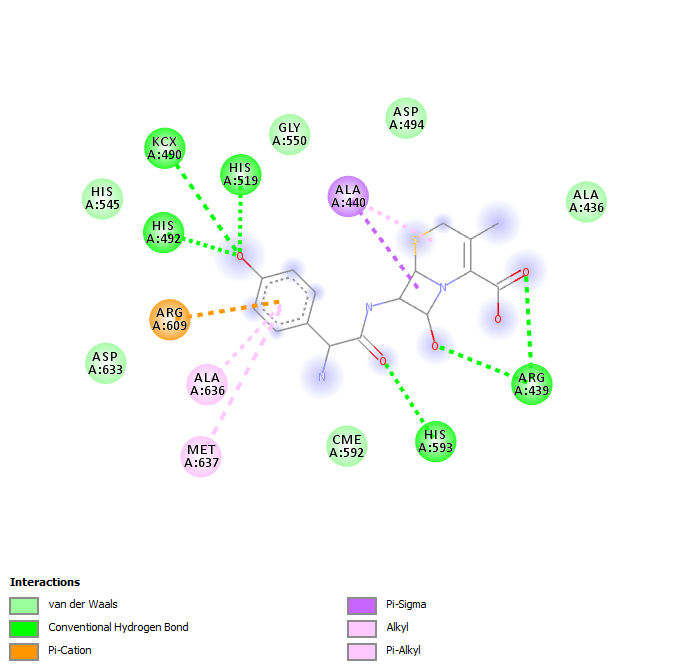
Cefalor Cefadroxil**


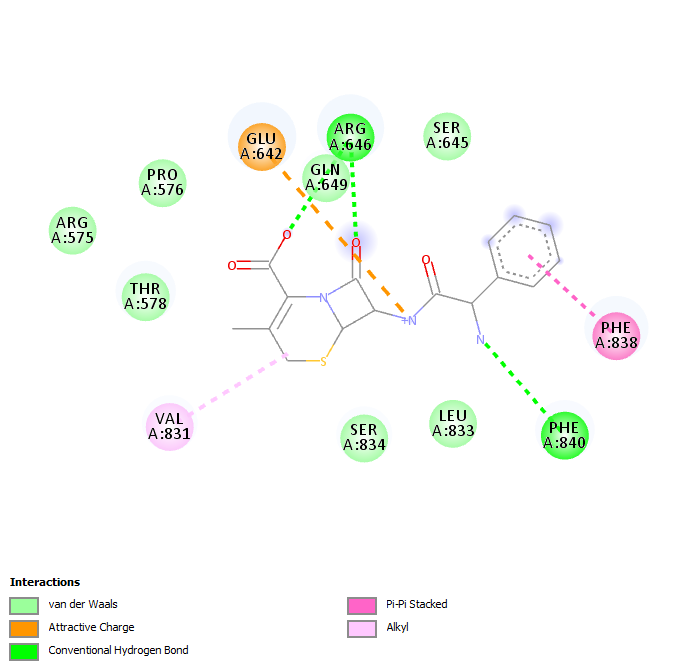


**Cefixime trihydrate** **Cefepime**


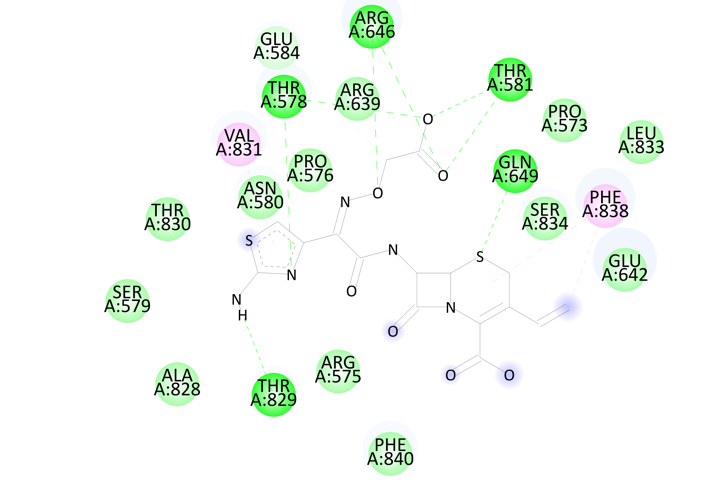

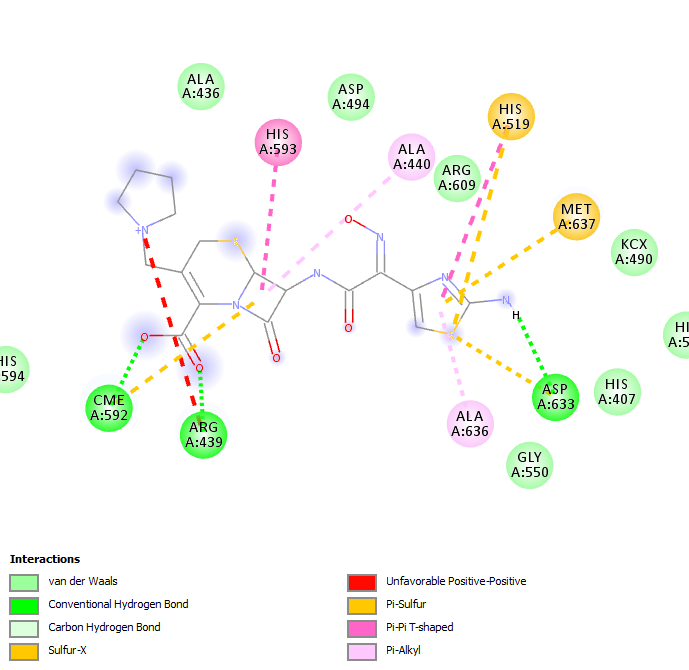


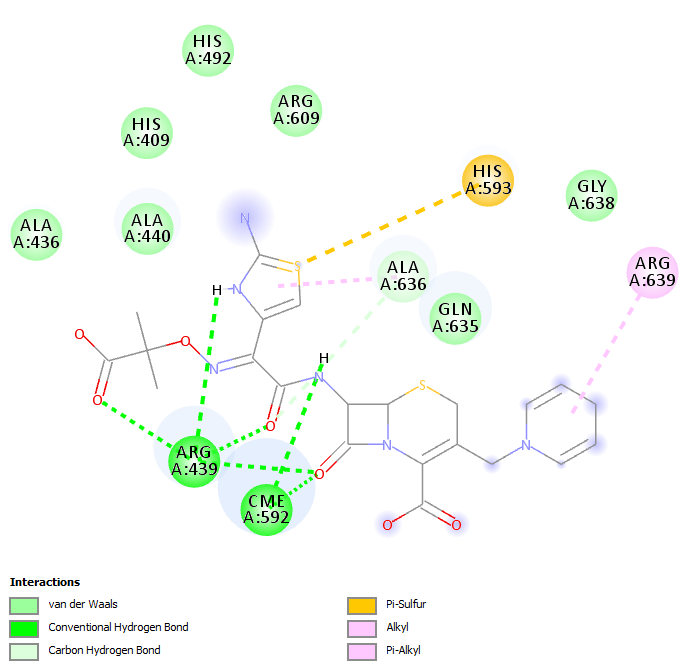

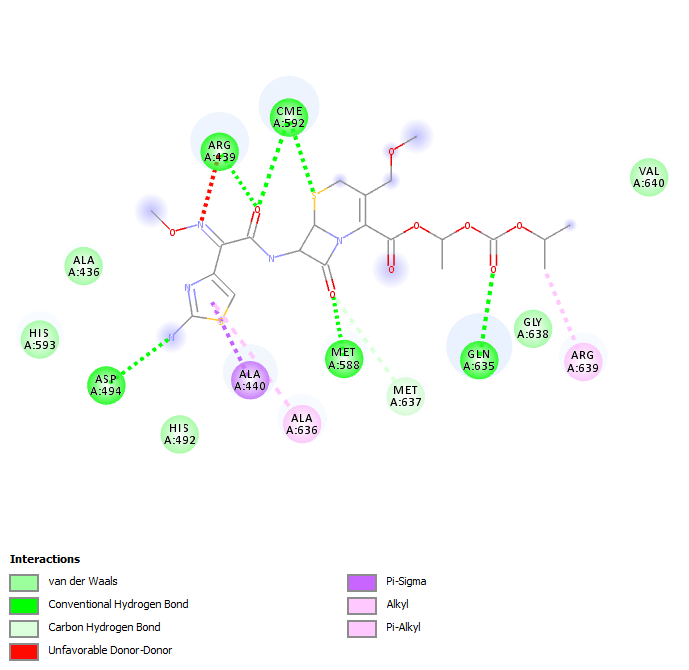
**Cefpodoxime proxetil**  **Ceftazidim**

**Cefuroxime**  **Cephalexin**


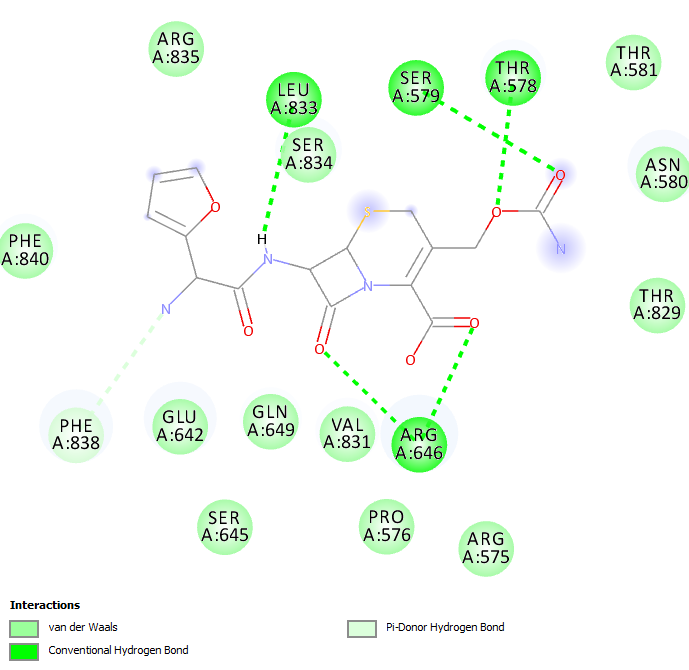

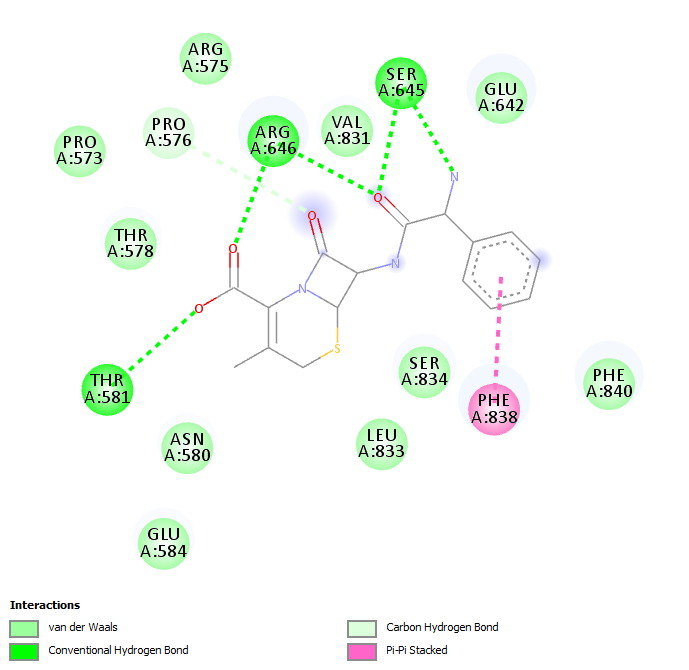


**Cephradine**


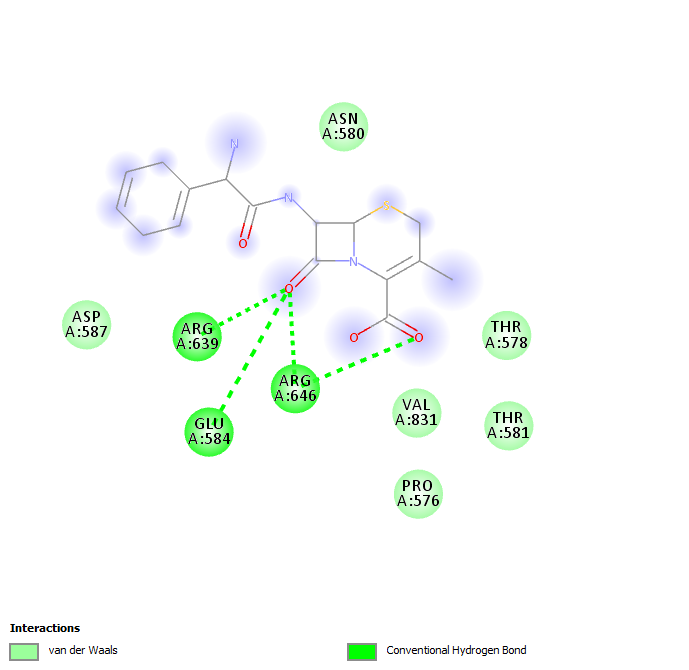


FIGURE S3. 2D docking of test drugs with urease enzyme.

**Cefalor Cefadroxil**

**
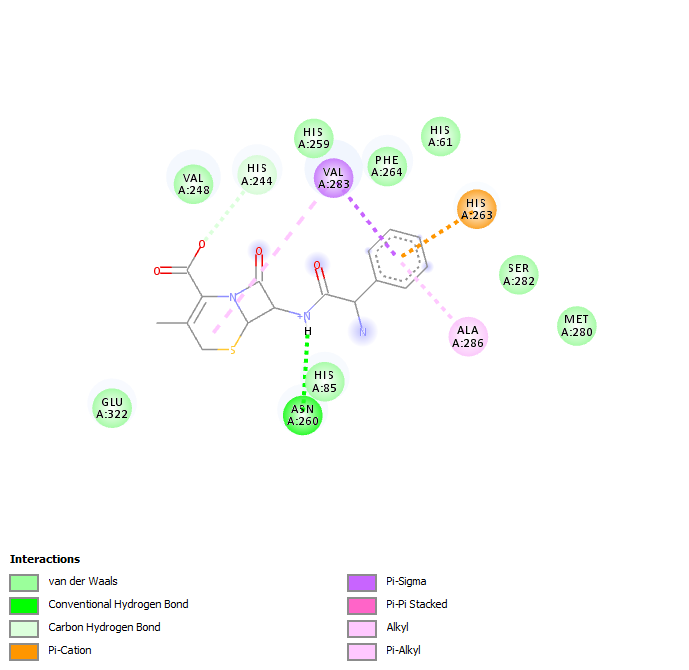

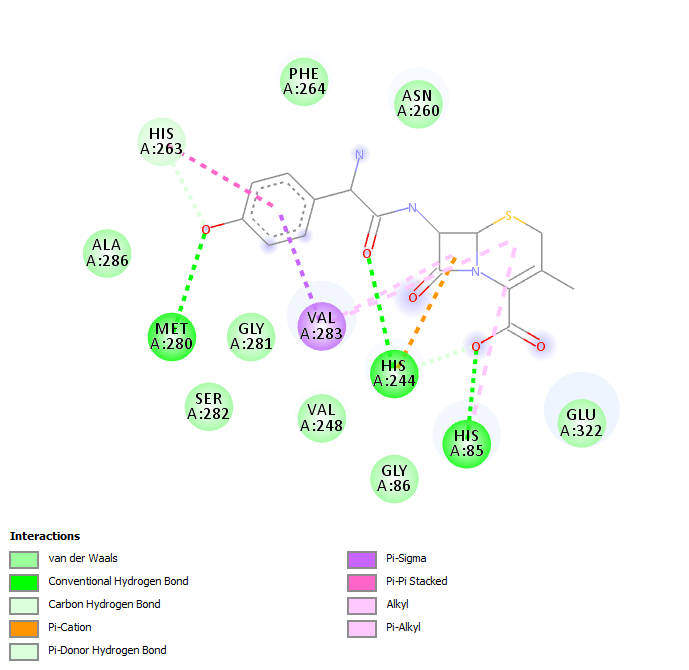
**

**Cefepime** **Cefixime trihydrate**

**
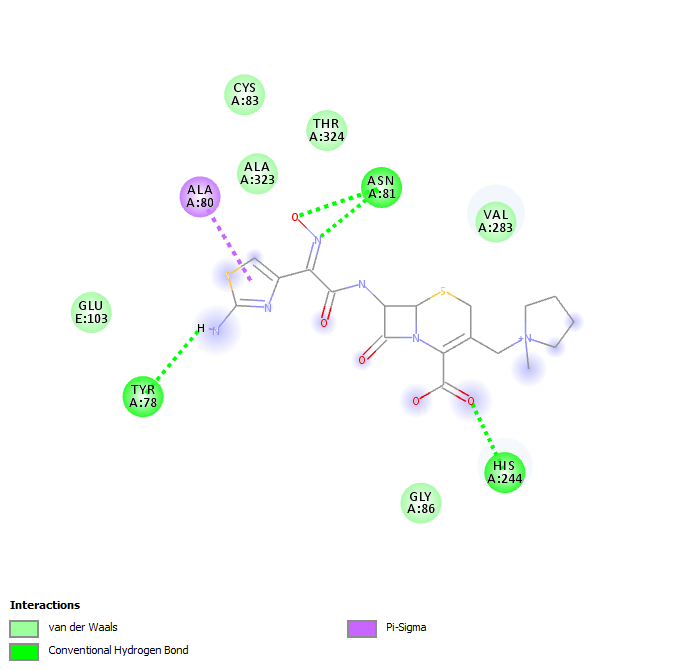
**
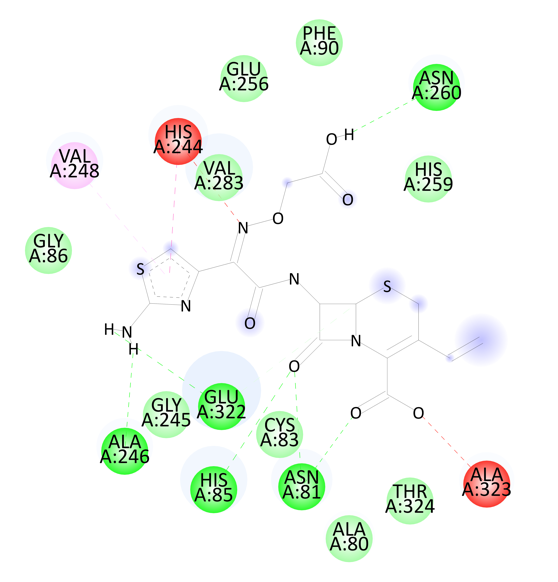


**Cefpodoxime proxetil**  **Ceftazidime**

**
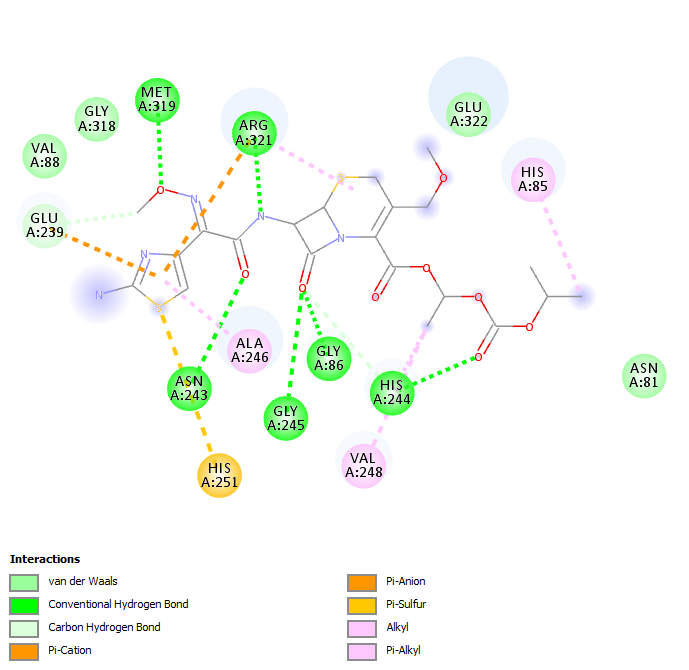

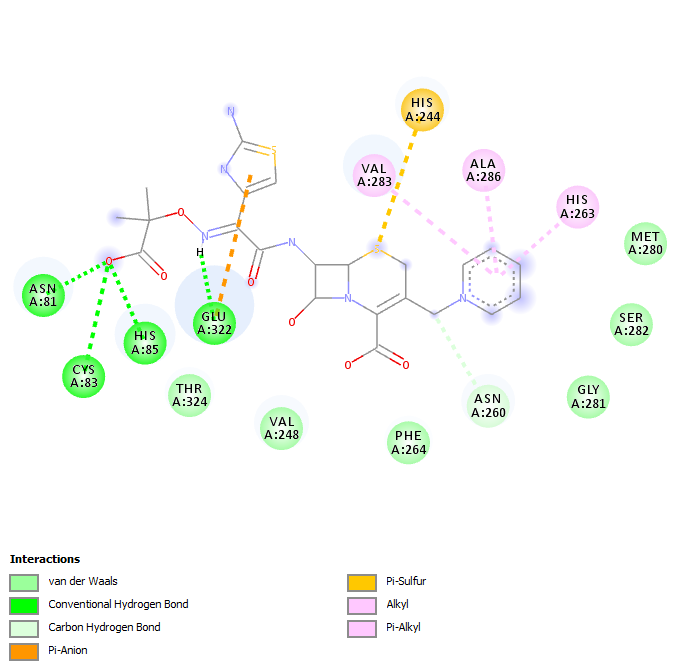
**

**Cefuroxime**  **Cephalexin**


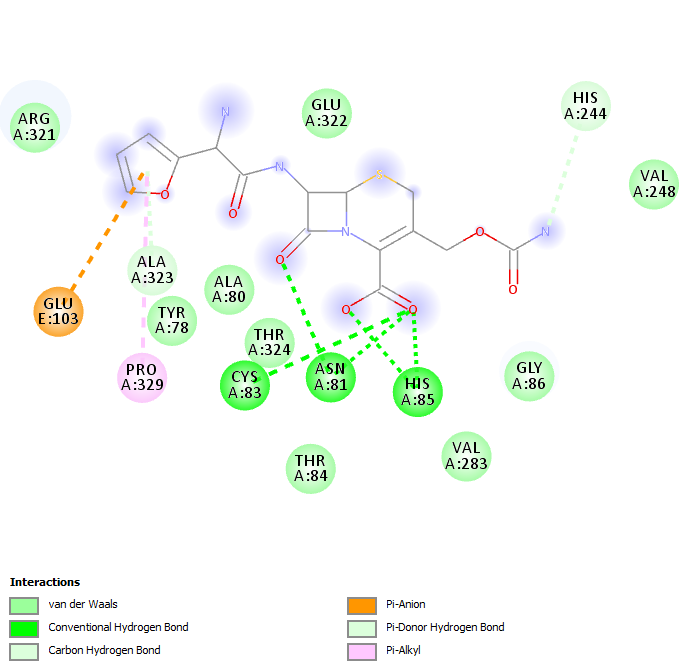

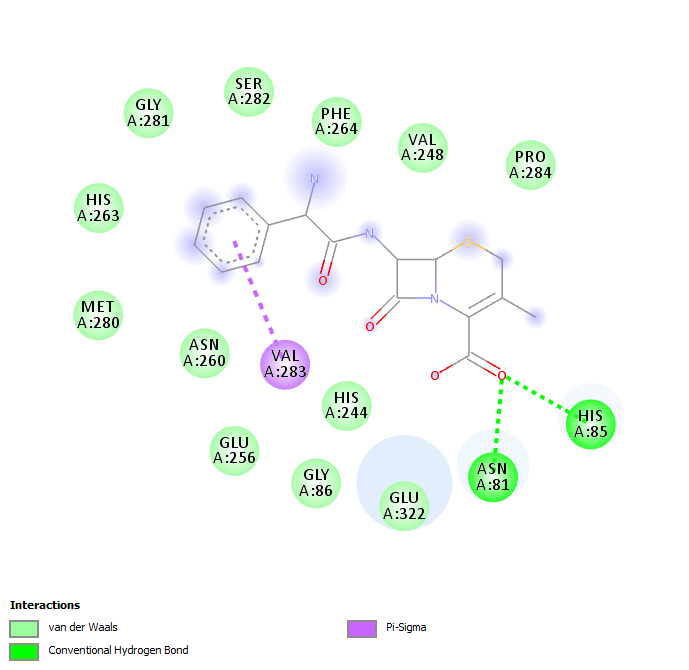


**Cephradine**


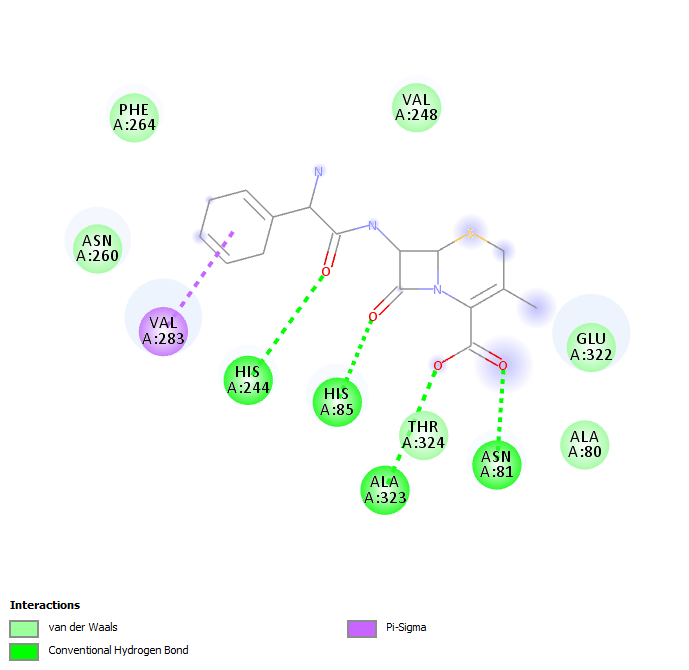


FIGURE S4. 2D docking of test drugs with tyrosinase enzyme.
